# Severe Agitation as an Unusual Presentation of Multisystem Inflammatory Syndrome in Children

**DOI:** 10.7759/cureus.27621

**Published:** 2022-08-02

**Authors:** Mohamed A Ghamry, Korlos Salib, Shereen Henin, Rehab Salah, Monica Dobs

**Affiliations:** 1 Pediatrics, Al Salam Hospital, Ministry of Health, Port Said, EGY; 2 General Practice, El-Demerdash Hospital, Cairo, EGY; 3 Internal Medicine/Pediatrics, California Institute of Behavioral Neurosciences and Psychology, Fairfield, USA; 4 Cardiology, Faculty of Medicine, Benha University, Benha, EGY; 5 Pediatrics, University of Florida College of Medicine, Jacksonville, USA

**Keywords:** covid-19, right coronary artery (rca), high fever, agitation, pediatric sepsis, pediatrics, post sars-cov-2 complication, altered mental state, multi-system inflammatory syndrome in children (mis-c)

## Abstract

Multisystem inflammatory syndrome in children (MIS-C) is a rare hyperinflammatory syndrome that mainly affects children after a primary infection with coronavirus disease 2019 (COVID-19), with the possibility of severe and lethal complications. We report a case of a unique presentation of MIS-C in a four-year-old boy who presented with severe agitation, muscle spasms, and two days of fever. Other findings consistent with MIS-C were revealed later, and he was managed with intravenous immunoglobulin (IVIG) and steroids. He showed a dramatic response of improvement and was discharged. This case report aimed to raise health professionals' awareness about the atypical presentations of MIS-C and the importance of early diagnosis, treatment, and follow-up MIS-C cases to avoid complications affecting children's lives.

## Introduction

Infection with coronavirus disease 2019 (COVID-19) has demonstrated a broad spectrum of clinical manifestations, ranging from asymptomatic to severe acute respiratory distress syndrome and death [1]. Although children are susceptible to COVID-19 infection as adults, they have a much lower rate of symptomatic primary infection [1]. However, a small percentage of children can develop multisystem inflammatory syndrome in children (MIS-C), which has been defined by the Centers for Disease Control and Prevention (CDC) and the World Health Organization [1]. The MIS-C diagnosis can be challenging due to its polymorphous presentation, so it requires a high index of suspicion to diagnose this syndrome quickly [1].

## Case presentation

A four-year-old male child with no past medical, psychiatric, or hospitalization history was admitted to the pediatric emergency service with severe agitation and muscle spasms as can be seen in Video 1.

**Video 1:** Severe agitation and muscle spasms shown in a case of child affected with "multisystem inflammatory syndrome in children" placed on unfit oxygen mask

**Figure 1:**
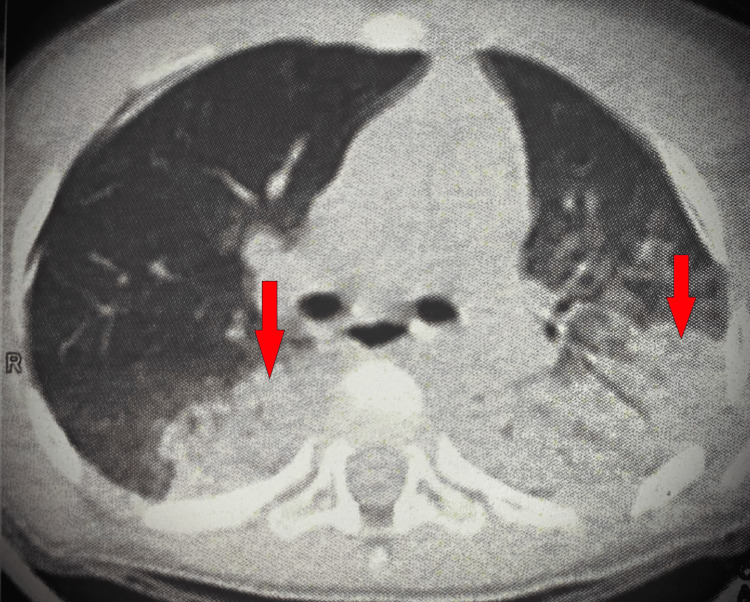
Chest CT showed bilateral lung opacities (arrows show examples of the opacities)

He was well two days ago when he developed a fever recorded up to 40°C, relieved with cold compresses and acetaminophen but returns every six hours. No history of recent illness, contact with a diseased person, or traveling. In the physical examination, he was alert, agitated, and febrile to 40°C with tachycardia and hypotension concerning shock. Laboratory studies are significant for low white blood cell count (2.9 k/μL) with low lymphocytes count (0.4 k/μL), high C-reactive protein (96 mg/dL), high creatinine (1.2 mg/dL), high blood urea nitrogen (30 mg/dL), elevated erythrocyte sedimentation rate (70 mm/h), raised ferritin (1330 ng/mL), high D-dimer (6 mg/L), and high CK-MB (137 IU/L). SARS-CoV-2 polymerase chain reaction testing was negative. Chest computed tomography (CT) showed bilateral lung opacities (Figure 1). He was placed on a non-rebreathing oxygen mask. He received fluid resuscitation, antipyretic, empiric antibiotics, and a pediatrics-neuropsychiatric consultation.

He was admitted to the pediatric intensive care unit, where he was placed on a high-flow nasal cannula and required initiation of norepinephrine to support falling blood pressure. After one hour, the patient develops attacks of muscle spasms and opisthotonos position with agitation and fluctuating level of consciousness. Soft wrist restraints were temporarily applied. Brain CT and magnetic resonance imaging showed no abnormalities. Upon thorough infection workup, the patient is confirmed to have COVID-19 immunoglobulin G (IgG)-positive, with the exclusion of other infections. Echocardiogram showed a left ventricular ejection fraction of 58% and mild right coronary artery (RCA) dilation (z-score < 2.5).

The patient met MIS-C CDC diagnostic criteria with clinical and laboratory findings. The patient was diagnosed with MIS-C, and treatment was started immediately. A single dose of intravenous immunoglobulin (IVIG) (2 g/kg), a five-day course of methylprednisolone (2 mg/kg/day), aspirin (4 mg/kg/day), and anticoagulation with low-molecular-weight heparin were started. Dramatic clinical response happened as the patient's fever, agitation, blood pressure, and level of consciousness improved to normal at the 12th hour after IVIG treatment, and norepinephrine was discontinued. In the follow-up, respiratory distress improved, inflammatory markers regressed, and RCA returned to normal in the echocardiogram. Antibiotherapy was discontinued on the eighth day of hospitalization, as no growth was detected in blood, cerebrospinal fluid, urine, and stool cultures. The patient was discharged at the end of the ninth day to continue aspirin therapy, and follow-up instructions were given.

## Discussion

A new entity termed MIS-C associated with COVID-19 was described by CDC in the United States on 14th May 2020 [2]. CDC defines MIS-C as a clinically severe illness requiring hospitalization in an individual aged <21 years proved to have current or recent SARS-CoV-2 infection or exposed to suspected or confirmed COVID-19 case within four weeks who presented with fever (>38.0°C for ≥24 hours), inflammatory marker elevation, and multisystem (>2) organ dysfunction, and in the absence of an alternative likely explanation [2].

MIS-C presentation involving four organ systems or more occurred in >70% of patients, with >50% admitted to an intensive care unit during their hospital stay (Table 1) [3]. Most common symptoms and signs are fever > 38°C (100%), gastrointestinal symptoms (53-92%), mucocutaneous involvement (70-74%), and hypotension (49-51%) [3]. Cardiac involvement is reported in 80% of MIS-C, including ventricular dysfunction, coronary artery dilation and aneurysms, arrhythmia, and conduction abnormalities [3,4]. Neuropsychiatric presentations were also reported, including irritability, headache, and confusion [5]. Hutchison et al. reported agitation and fluctuating awareness levels during an adolescent boy's MIS-C disease course. However, Hutchison et al. suggested multiple explanations for these symptoms. They then outweighed the probability that the effect of high-dose corticosteroid therapy may cause these symptoms because the neuropsychiatric symptoms had appeared after initiating it and disappeared after discontinuing it [5]. In this case report, we report agitation as an initial presentation of MIS-C with a fluctuating level of consciousness before corticosteroid therapy initiation, which excludes that it may be secondary to corticosteroid therapy. The effect of COVID-19 infection or MIS-C on the nervous system remains unclear, and the neurological manifestations may be primary, secondary, or both [5].

**Table 1:** Comparison between presenting clinical symptoms in children with MIS-C around the world MIS-C: multisystem inflammatory syndrome in children

Presenting symptoms (N)	The median age in years	Total number of cases	Country	Reference
Fever (10), conjunctivitis (5), rash (5), cervical lymphadenopathy (7), mucosal changes (4), diarrhea (6)	7.5	10	Italy	Verdoni et al. [6]
Fever (3), cough (2), dyspnea (2), sore throat (2), vomiting (2), abdominal pain (2)	11	3	Switzerland	Dallan et al. [7]
Fever, rash, irritability, red lips	0.3	1	India	Acharrya et al. [8]
Fever (21), Rash (15), lip changes (15), conjunctivitis (17), diarrhea (20), vomiting (20), abdominal pain (21), irritability (12), headache (6), confusion (6)	7.9	21	France	Toubiana et al. [9]
Fever (58), abdominal pain (31), diarrhea (30), rash (30), vomiting (26), conjunctivitis (26), headache (15), respiratory symptoms (12), lymphadenopathy (9), swollen hands and feet (9), sore throat (6), confusion (5)	9	58	United Kingdom	Whittaker et al. [10]
Fever (99), chest pain (11), abdominal pain (60), nausea or vomiting (57), diarrhea (49), rash (59), swollen hands or feet (9), conjunctivitis (55), mucosal changes (27), headache (29), confusion or altered mental status (2), musculoskeletal pain (20), sore throat (16), congestion (13), cough (31), dyspnea (19), wheezing (1)	0-20	99	United States of America	Dufort et al. [11]
Fever, agitation, muscle spasms, fluctuating level of consciousness	4	1	Egypt	This study

IVIG has been shown to be effective in treating MIS-C. Corticosteroids can be used based on the case and the followed guidelines. However, MIS-C management is multidisciplinary, and treatment should be individualized according to the system involved [12]. Long-term follow-up is essential, especially in children who have experienced complications [12]. The successful response for IVIG in treating MIS-C and Kawasaki disease suggests that antibodies likely have a role in the pathophysiology, but the exact role of antibodies is still unknown [13].

## Conclusions

MIS-C is a serious complication of COVID-19 infection, and health care providers should have a high index of suspicion for MIS-C, especially if multiple systems are affected, history of infection or exposure to COVID-19, laboratory evidence of COVID-19 infection or antibodies, or unusual presentations in a feverish child. Neuropsychiatric manifestations can be the first presentation of this syndrome. Further studies about the pathogenesis of MIS-C will be beneficial in understanding the nature of the disease. As this is a new pediatric syndrome, further research and case reports are needed to assist other clinicians to early diagnose the disease and contribute to the most effective preventive and therapeutic strategies to save children's lives and keep them healthy.
